# The association between the apolipoprotein B/A-I ratio and coronary calcification may differ depending on kidney function in a healthy population

**DOI:** 10.1371/journal.pone.0185522

**Published:** 2017-09-28

**Authors:** Seok-hyung Kim, Donghwan Oh, Kwon Soo Jung, Jung Eun Lee, Hyunwook Kim, Hyung Jong Kim, Beom Seok Kim, Hyeong Cheon Park, Byoung Kwon Lee, Hoon Young Choi

**Affiliations:** 1 Department of Internal Medicine, Gangnam Severance Hospital, Yonsei University College of Medicine, Seoul, Korea; 2 Department of Internal Medicine, Yongin Severance Hospital, Yonsei University College of Medicine, Seoul, Korea; 3 Department of Internal Medicine, CHA Bundang Medical Center, CHA University, Seongnam, Korea; 4 Department of Internal Medicine, Severance Hospital, Yonsei University College of Medicine, Seoul, Korea; 5 Severance Institute for Vascular and Metabolic Research, Yonsei University College of Medicine, Seoul, Korea; Heart Research Institute, AUSTRALIA

## Abstract

**Background:**

The apolipoprotein B/A-1 ratio has been reported to be one of the strongest risk predictors of cardiovascular events. However, its prognostic value for cardiovascular disease is still uncertain, especially in patients with chronic kidney disease. This study aimed to investigate whether the association between the apolipoprotein B/A-I ratio and coronary artery calcification differed according to kidney function in a healthy population.

**Methods:**

Of the data from 7,780 participants from the medical records database in Gangnam Severance Hospital from 2005 through 2016, a cross-sectional analysis included participants with an estimated glomerular filtration rate (eGFR) ≥ 60 mL/min/1.73 m^2^ determined based on the Chronic Kidney Disease -Epidemiology Collaboration equation (n  =  1,800). Mild renal insufficiency was defined as an eGFR of 60–90 mL/min/1.73 m^2^. Coronary artery calcification measured with computed tomography was defined as an above-zero score. Logistic regression analyses were used to determine the association between coronary calcification and the apolipoprotein B/A-I ratio according to eGFR by adjusting for the influence of confounders.

**Results:**

The mean apolipoprotein B/A-I level was significantly higher in the participants with coronary artery calcification than in the participants without coronary artery calcification. The apolipoprotein B/A-I ratio was significantly different according to coronary artery calcification in the participants with normal kidney function, but in the participants with mild renal insufficiency, it was not different. After adjusting for age, male sex, systolic blood pressure, body mass index, current smoking status, and fasting plasma glucose, the apolipoprotein B/A-I ratio was significantly associated with an increased risk of coronary artery calcification in participants with normal kidney function (odds ratio = 2.411, *p* = 0.011), while in the participants with mild renal insufficiency, the apolipoprotein B/A-I ratio was not associated with coronary artery calcification.

**Conclusion:**

Our study showed that the predictive value of apolipoprotein B/A-I ratio for coronary artery calcification may differ according to kidney function.

## Introduction

Cardiovascular disease (CVD) is a major cause of death worldwide, and its contribution to the disease burden is expected to increase over the next 15 years [1, 2]. Therefore, efforts to identify cardiovascular risk factors have been made worldwide.

Apolipoproteins are important proteins in lipoprotein particles [3]. Apolipoprotein A-I (apoA-I) is a major apolipoprotein associated with high-density lipoprotein (HDL)-cholesterol, while apolipoprotein B (apoB) exists as a single molecule in all potentially atherogenic lipoprotein particles [4]. Therefore, the ratio of apoB/A-I may reflect the cholesterol balance between potentially atherogenic and antiatherogenic lipoprotein particles. The apoB/A-I ratio has been reported to be a better predictor of cardiovascular risk than routine clinical lipid measurements in recent clinical studies [5–7]. However, data are still conflicting, especially in patients with chronic kidney disease (CKD) [8, 9].

Decreased renal function is associated with many disruptions in lipoprotein metabolism, leading to dyslipidemia and accumulation of atherogenic particles [10, 11]. Apolipoproteins have been reported to be associated with renal function [12, 13]. A cross-sectional analysis of two independent general population-based studies showed that a higher serum apoA-I level was associated with a lower prevalence of CKD, and a lower serum apoA-I level and a higher apoB/A-I ratio were associated with a lower estimated glomerular filtration rate (eGFR) [12]. However, an association between apolipoproteins and cardiovascular risk in patients with CKD was not found. Although a lower apoA-I concentration and a higher apoB/A-I ratio were found in patients with CKD, there was no significant evidence that these apolipoproteins were more strongly associated with coronary heart disease incidence in CKD compared to other lipid measurements routinely measured in clinical settings [9]. Given these discordant results, the association between apolipoproteins and CVD is still controversial. Furthermore, this association has been relatively underexplored in healthy participants.

The present study investigated whether apolipoproteins were associated with coronary artery calcification (CAC) in healthy participants after adjustment for other atherosclerosis risk factors and whether this association differs according to renal function status. For this study, only asymptomatic individuals who underwent extensive cardiac risk factor evaluation were enrolled to determine the relationship between apoB/A-I levels and the CAC score in healthy participants considering multiple confounding factors.

## Materials and methods

### Participants

**Fig 1:**
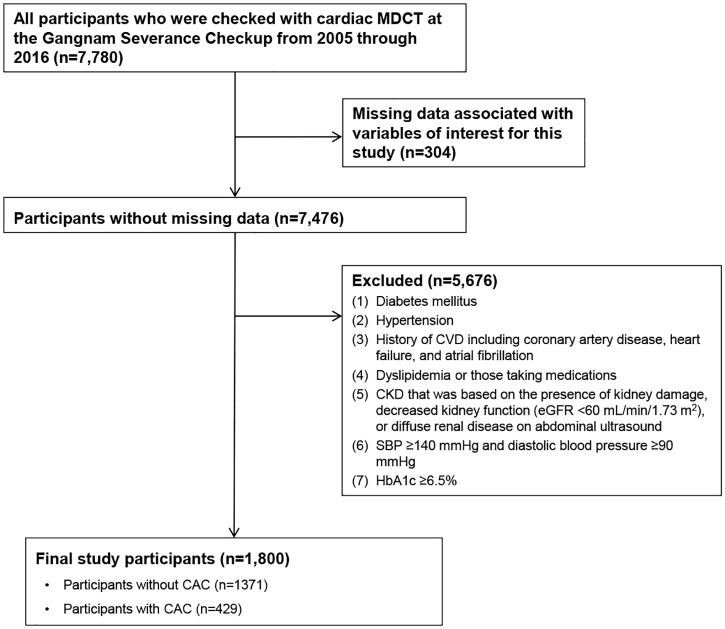
Diagram of participant enrollment. CAC: coronary artery calcification CKD: chronic kidney disease; CVD: cardiovascular disease; eGFR: estimated glomerular filtration rate; MDCT: multidetector computed tomography.

This retrospective cross-sectional study included adult participants who underwent a routine health examination at Gangnam Severance Hospital from 2005 through 2016. Data of a total of 7,780 participants who had CAC measured were collected from the medical records database from Gangnam Severance Hospital. There were 346 patients with missing data associated with the variables of interest. The study excluded an additional 5,676 participants who had (1) diabetes mellitus; (2) hypertension; (3) a history of CVD, including coronary artery disease, heart failure, and atrial fibrillation; (4) dyslipidemia or took medications such as statins or fenofibrate; or (5) CKD that was based on the presence of kidney damage (dipstick proteinuria of 1+, 2+, 3+, or 4+), decreased kidney function (eGFR <60 mL/min/1.73 m^2^), or diffuse renal disease on an abdominal ultrasonogram. Furthermore, participants with systolic blood pressure (SBP) ≥140 mmHg, diastolic blood pressure ≥90 mmHg, or glycosylated hemoglobin level ≥6.5% were excluded. Finally, 1,800 participants were included in this study (Fig 1).

The Institutional Review Board of Gangnam Severance Hospital, Yonsei University College of Medicine, approved the study protocol (no. 3-2016-0061), and the need for informed consent was waived owing to the retrospective nature of the study.

### Clinical and laboratory data

We reviewed the patients’ medical records, including age, sex, height, body weight, current smoking status, and blood pressure, which were measured during the visits. Blood samples were obtained on the day that cardiac computed tomography was performed, after a fasting period of at least 12 hours. The following laboratory parameters were measured: blood urea nitrogen (BUN), creatinine, fasting glucose, low-density lipoprotein (LDL)-cholesterol, high-density lipoprotein (HDL)-cholesterol, apoB, and apoA-I. LDL and HDL-cholesterol were measured directly by a homogenous enzymatic colorimetric assay using a Beckman Coulter AU5800 analyzer (Beckman Coulter Inc., Brea CA, USA). Serum apoB and apoA-I concentrations were measured by an immunoturbidimetric method using a Beckman Coulter AU5800 analyzer [14]. Body mass index (BMI) was calculated as body weight (kilograms) divided by the square of height (square meters). Participants were classified as current smokers or non-smokers based on their smoking habits. Kidney function was assessed based on the eGFR that was calculated using the Chronic Kidney Disease Epidemiology Collaboration (CKD-EPI) equation based on serum creatinine levels [15]. Mild renal insufficiency (RI) was defined as an eGFR of 60–90 mL/min/1.73 m^2^ [16, 17].

Participants were scanned using cardiac multidetector computed tomography (Philips Brilliance 64; Philips Medical Systems, Best, The Netherlands) with a 3-mm slice thickness and 1.5-mm reconstruction interval. Participants with a heart rate over 66 beats/min took beta-blocking agents (25 mg atenolol; Tenormin, Hyundai, Seoul, Korea) before CT scanning. The CAC score was measured using the Agatston method [18, 19]. CAC scores above zero indicated CAC [20].

### Statistical analysis

Data are presented as mean ± standard deviation for continuous variables or a frequency (percentage) for categorical variables. Statistical differences in the clinical characteristics between the two groups were determined using the independent t-test for continuous variables and the chi-square test for categorical variables. A comparative analysis of four groups according to kidney function and CAC status was performed with analysis of variance test. *P*-values of post-hoc analysis were corrected by Bonferroni correction.

Univariate and multivariate logistic regression analyses were used to determine the association between CAC and the apoB/A-I ratio according to the eGFR status by adjusting for the influence of confounders. The predictive value of the apoB/A-I ratio for CAC was calculated by constructing receiver operating characteristic (ROC) curves. *P*-values <0.05 were considered statistically significant.

All analyses were conducted using the Statistical Package for the Social Sciences (SPSS^®^) version 20.0 (IBM Corp., Armonk, NY, USA).

## Results

### Participants’ characteristics

The clinical characteristics of the participants are shown in Table 1. The 1,800 healthy participants who were included had a mean age of 52.1 ± 9.5 years. The mean eGFR level was 97.5 ± 12.1 mL/min/1.73 m^2^. The clinical characteristics and biochemical findings of patients without and with CAC are also shown in Table 1. Age, the proportion of male sex, BMI, SBP, proportion of current smokers, fasting glucose, and LDL-cholesterol levels were significantly higher in participants with CAC than in those without CAC. Participants with CAC had significantly lower eGFR and HDL-cholesterol levels than participants without CAC. ApoB was significantly higher and apoA-I was lower in participants with CAC than in those without CAC. The apoB/A-I ratio was significantly higher among participants with CAC (0.78 ± 0.23 vs. 0.72 ± 0.23, *P* < 0.001) (Table 1).

**Table 1:** Characteristics of study participants classified by CAC.

Clinical variables	All participants (n = 1800)	Participants without CAC (n = 1371)	Participants with CAC (n = 429)	*P* -value
Age (years)	52.1 ± 9.5	50.3 ± 9.0	58.0 ± 8.8	<0.001
Sex				<0.001
Female (n (%))	552(30.7)	504(36.8)	48(11.2)	
Male (n (%))	1248(69.3)	867(63.2)	381(88.8)	
BMI (kg/m^2^)	23.9 ± 3.2	23.7 ± 3.2	24.5 ± 2.9	<0.001
SBP (mmHg)	120.9 ± 11.8	120.2 ± 11.9	123.2 ± 11.5	<0.001
Current smoker (n (%))	409 (21.7)	285 (20.8)	109 (25.4)	0.045
Fasting glucose (mg/dL)	96.8 ± 14.0	95.5 ± 13.2	101.0 ± 15.5	<0.001
eGFR (mL/min/1.73 m^2^)	97.5 ± 12.1	99.0 ± 11.8	92.8 ± 11.7	<0.001
HDL-cholesterol (mg/dL)	50.1 ± 12.9	50.8 ± 13.2	48.1 ± 11.5	<0.001
LDL-cholesterol (mg/dL)	130.7 ± 34.1	129.8 ± 33.9	133.6 ± 34.5	0.043
Apo A-I (mg/dL)	142.4 ± 24.2	143.2 ± 24.6	139.8 ± 22.9	0.013
Apo B (mg/dL)	101.4 ± 24.3	100.0 ± 24.4	106.0 ± 23.8	<0.001
Apo B/A-I ratio	0.74 ± 0.23	0.72 ± 0.23	0.78 ± 0.23	<0.001

Data are presented as mean ± standard deviation or number (percentage) of subjects. CAC, coronary artery calcification; BMI, body mass index; SBP, systolic blood pressure; eGFR, estimated glomerular filtration rate; HDL, high-density lipoprotein; LDL, low-density lipoprotein; apo, apolipoproteins

The independent t-test for continuous variables and the chi-square test for categorical variables were performed to compare the two groups.

### Association of the CAC score with the apoB/A-I ratio according to kidney function

Table 2 shows the clinical parameters of participants with normal kidney function according to CAC. In participants with normal kidney function, age, the proportion of male sex, BMI, SBP, fasting glucose, and LDL-cholesterol levels were significantly higher, whereas the eGFR and HDL-cholesterol level were lower in those with CAC than in those without CAC. ApoA-I was also significantly lower (apoA-I: 138.5 ± 20.8 vs. 99.8 ± 24.7 mg/dL, *P* = 0.001, while apoB and the apoB/A-I ratio were higher in participants with CAC than in those without CAC (apoB: 107.5 ± 23.6 vs. 99.8 ± 24.7 mg/dL, *P <* 0.001, apoB/A-I ratio: 0.80 ± 0.22 vs. 0.72 ± 0.23, *P <* 0.001) (Table 2).

**Table 2:** Comparison of clinical characteristics of participants with normal kidney function by CAC.

Clinical variables	No CAC (n = 1081)	CAC (n = 269)	*p*-value
Age (years)	49.1 ± 8.6	55.5 ± 7.7	<0.001
Sex			<0.001
Female (n (%))	88(30.34)	24(15)	
Male (n (%))	202(69.66)	136(85)	
BMI (kg/m^2^)	23.6 ± 3.3	24.3 ± 2.9	0.001
SBP (mmHg)	120.2 ± 11.8	123.4 ± 11.4	<0.001
Current smoker (n (%))	236 (21.8)	73 (27.1)	0.074
Fasting glucose (mg/dL)	95.2 ± 13.4	101.1 ± 15.9	<0.001
eGFR (mL/min/1.73 m^2^)	103.7 ± 7.9	100.1 ± 6.9	<0.001
HDL-cholesterol (mg/dL)	50.9 ± 13.3	47.6 ± 10.9	<0.001
LDL-cholesterol (mg/dL)	127.9 ± 33.9	134.7 ± 35.3	0.003
Apo A-I (mg/dL)	143.2 ± 24.9	138.5 ± 20.8	0.001
Apo B (mg/dL)	99.8 ± 24.7	107.5 ± 23.6	<0.001
Apo B/A-I ratio	0.72 ± 0.23	0.80 ± 0.22	<0.001

Data are presented as mean ± standard deviation or number (percentage) of subjects. CAC, coronary artery calcification; BMI, body mass index; SBP, systolic blood pressure; eGFR, estimated glomerular filtration rate; HDL, high-density lipoprotein; LDL, low-density lipoprotein; apo, apolipoproteins. The independent t-test for continuous variables and the chi-square test for categorical variables were performed to compare the two groups.

Similar to these results, in participants with mild RI, age, the proportion of male sex, BMI, SBP, and fasting glucose level were also significantly higher in participants with CAC than in participants without CAC. However, apoA-I, apoB, and the apoB/A-I ratio were not significantly different among participants with or without CAC (Table 3).

**Table 3:** Comparison of clinical characteristics of participants with mild RI by CAC.

Clinical variables	No CAC (n = 290)	CAC (n = 160)	*p*-value
Age (years)	54.7 ± 8.8	62.2 ± 8.9	<0.001
Sex			<0.001
Female (n (%))	88(30.34)	24(15)	
Male (n (%))	202(69.66)	136(85)	
BMI (kg/m^2^)	24.0 ± 2.9	24.7 ± 2.9	0.026
SBP (mmHg)	120 ± 12.0	122.9 ± 11.6	0.019
Current smoker (n (%))	49 (16.9)	36 (22.5)	0.167
Fasting glucose (mg/dL)	96.8 ± 12.4	100.7 ± 14.8	0.003
eGFR (mL/min/1.73 m^2^)	81.6 ± 6.5	80.4 ± 7.0	0.084
HDL-cholesterol (mg/dL)	50.2 ± 12.8	48.8 ± 12.5	0.281
LDL-cholesterol (mg/dL)	137.1 ± 32.6	131.8 ± 33.2	0.102
Apo A-I (mg/dL)	142.8 ± 23.6	142.1 ± 25.9	0.762
Apo B (mg/dL)	100.7 ± 23.2	103.5 ± 23.9	0.232
Apo B/A-I ratio	0.73 ± 0.22	0.76 ± 0.24	0.183

Data are presented as mean ± standard deviation or number (percentage) of subjects. RI, renal insufficiency; CAC, coronary artery calcification; BMI, body mass index; SBP, systolic blood pressure; eGFR, estimated glomerular filtration rate; HDL, high-density lipoprotein; LDL, low-density lipoprotein; apo, apolipoproteins. The independent t-test for continuous variables and the chi-square test for categorical variables were performed to compare the two groups.

We also compared four groups according to CAC and the kidney function status using Bonferroni correction. The apoB/A-I ratio according to CAC status was not significantly different in the participants with mild RI (S1 Table).

Univariate logistic regression analysis to examine the association of individual study covariates with CAC identified the risk factors that were included in multivariate models. Age, male sex, BMI, SBP, current smoking status, fasting glucose level, HDL cholesterol level, LDL cholesterol level, and central SBP significantly affected CAC in all participants. Moreover, the apoB/A-I ratio was a significant risk factor affecting CAC (odds ratio [OR] 3.026 (1.892–4.840), *P* < 0.001) (Table 4).

**Table 4:** Univariate logistic regression analysis to determine the independent factors affecting CAC.

Variables	OR (95% CI)	*P-* value
Age	1.104 (1.088–1.120)	< 0.001
Male	4.614 (3.352–6.352)	< 0.001
BMI	1.077 (1.041–1.114)	<0.001
SBP	1.023 (1.013–1.033)	<0.001
Current smoker	1.298 (1.007–1.672)	0.044
Fasting glucose	1.026 (1.019–1.034)	<0.001
HDL-cholesterol	0.982 (0.974–0.991)	<0.001
LDL-cholesterol	1.003 (1.000–1.006)	0.043
Apo B/A-I ratio	3.026 (1.892–4.840)	<0.001

CAC, coronary artery calcification; SBP, systolic blood pressure; BMI, body mass index; HDL, high-density lipoprotein; LDL, low-density lipoprotein; eGFR, estimated glomerular filtration rate; OR: odds ratio; CI: confidence interval

Next, multivariate logistic regression analysis was performed for CAC as a dependent variable. We adjusted for CVD risk factors such as age, sex, BMI, SBP, current smoking status, and fasting serum glucose to investigate the effects of the apoB/A-I ratio on CAC. To compare the apoB/A-I ratio to other lipid measurements, including HDL-cholesterol and LDL-cholesterol, we also performed multivariate logistic regression analysis using HDL-cholesterol and LDL-cholesterol as dependent variables for CAC. In model 1, we investigated the association of CAC with the apoB/A-I ratio and other lipid measurements after adjusting for age and sex. The apoB/A-I ratio and LDL cholesterol levels were independently associated with CAC after adjustment for age and sex in all participants (apoB/A-I ratio: OR 2.092 (1.213–3.610), *P* = 0.008, LDL-cholesterol: OR 1.005 (1.001–1.009), *P* = 0.004) and in participants with normal kidney function (apoB/A-I ratio: OR 2.635 (1.358–5.113), *P* = 0.004, LDL-cholesterol: OR 1.008 (1.004–1.013), *P <* 0.001). However, in participants with mild RI, the apoB/A-I ratio and other lipid measurements were not associated with CAC (Table 5). In model 2, we included classic atherosclerosis risk factors such as BMI, SBP, current smoking status, and fasting glucose level in addition to the variables included in model 1. In all participants and participants with normal kidney function, the apoB/A-I ratio and LDL-cholesterol level were significantly associated with CAC in model 2 (apoB/A-I ratio: OR 1.818 (1.039–3.181), *P* < 0.001, LDL-cholesterol: OR 1.005 (1.001–1.008), *P* = 0.011, in all participants; apoB/A-I ratio: OR 2.411 (1.224–4.748), *P* = 0.011, LDL-cholesterol: OR 1.008 (1.004–1.012), *P* < 0.001, in participants with normal kidney function) (Table 4). Similar to model 1, in participants with mild RI, the apoB/A-I ratio and other lipid measurements were not associated with CAC in model 2 (Table 5).

**Table 5:** Multivariate logistic regression analysis for CAC according to kidney function.

	All participants		Participants with normal kidney function		Participants with mild RI	
Variables	OR (95% CI)	*P-* value	OR (95% CI)	*P-* value	OR (95% CI)	*P* -value
***Model 1***						
HDL-cholesterol	1.002(0.991–1.013)	0.743	0.999 (0.986–1.012)	0.826	1.008 (0.989–1.027)	0.406
LDL-cholesterol	1.005 (1.001–1.009)	0.004	1.008 (1.004–1.013)	<0.001	0.998 (0.992–1.005)	0.584
Apo B/A-I ratio	2.092 (1.213–3.610)	0.008	2.635 (1.358–5.113)	0.004	1.369 (0.521–3.593)	0.524
***Model 2***						
HDL-cholesterol	1.005 (0.994–1.016)	0.362	1.001 (0.987–1.014)	0.940	1.015 (0.995–1.035)	0.148
LDL-cholesterol	1.005 (1.001–1.008)	0.011	1.008 (1.004–1.012)	<0.001	0.998 (0.991–1.004)	0.479
Apo B/A-I ratio	1.818 (1.039–3.181)	<0.001	2.411 (1.224–4.748)	0.011	1.074 (0.395–2.925)	0.888

Model 1, adjusted by age and sex; Model 2, adjusted by age, sex, BMI, SBP, current smoker, fasting glucose

CAC, coronary artery calcification; RI, renal insufficiency; SBP, systolic blood pressure; BMI, body mass index; HDL, high-density lipoprotein; LDL, low-density lipoprotein; OR: odds ratio; CI: confidence interval

**Fig 2:**
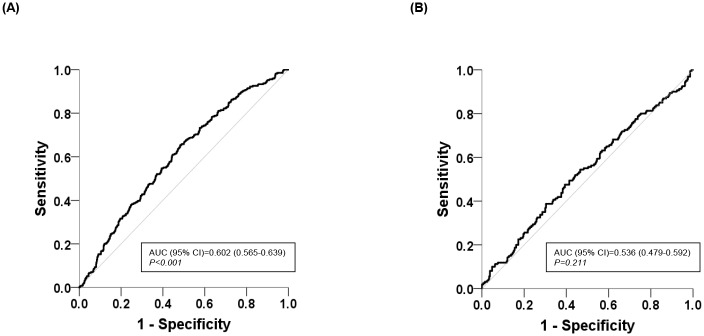
ROC curves for coronary artery calcification based on the serum apolipoprotein B/A-I ratio. (A) ROC curve in participants with normal kidney function, (B) ROC curve participants with mild RI. ROC: receiver operating characteristic, AUC: area under the curve; CI: confidence interval.

We also constructed ROC curves to predict CAC based on apo B/A-I levels according to kidney function status. The area under the ROC curve (AUC) of apoB/A-I levels for CAC in participants with normal kidney function was larger than that in participants with mild RI (AUC in participants with normal kidney function = 0.602; 95% confidence interval [CI], 0.565–0.639, *P* < 0.001; Fig 2A vs. AUC in participants with mild RI = 0.536; 95% CI, 0.479–0.592, *P* = 0.211; Fig 2B).

## Discussion

The present study assessed the association between the serum apoB/A-I ratio and CAC according to kidney function status in a large number of healthy subjects without a previous history of CVD or other confounding risk factors for CVD, including diabetes, hypertension, or dyslipidemia. Serum apoB/A-I ratios may not be helpful to predict CAC in participants with mild RI (90–60 mL/min/1.73 m^2^ of eGFR), although these participants did not have CKD, which is defined as impaired kidney function (less than 60 mL/min/1.73 m^2^ of eGFR) or evidence of kidney damage.

CVD correlates with kidney function decline, and its risk has been reported to increase early in the natural history of CKD [21–24]. Mild RI, which was a decrease in kidney function from 90 to 60 mL/min/1.73 m^2^ of eGFR, was associated with a four-fold increased risk of cardiovascular death. This association between mild RI and cardiovascular mortality cannot be explained by the presence of hypertension, diabetes, or prior cardiovascular disease, the lipid profile, the homocysteine level, or by markers of endothelial dysfunction or inflammation. The mechanism responsible for the association thus remains unclear [25].

Apolipoproteins have been shown to be strong independent predictors of CVD in the general population [9, 26, 27]. Several studies have indicated that apolipoproteins are better predictors of the risk of atherosclerotic risk than the traditional lipid parameters currently used in clinical practice, either separately or calculated together as the apoB/A-I ratio [6]. In a large population without a previous history of CVD, serum apoB/A-I ratios were significantly associated with coronary artery stenosis, as determined using multidetector computed tomography. An increase in the serum apoB/A-I ratio was associated with significant coronary atherosclerosis independent of conventional risk factors [28]. Moreover, apolipoprotein levels at a young age may predict midlife CAC better than LDL-C and non-HDL-C independent of baseline traditional CVD risk factors [29]. The apoB/A-I ratio is closely associated with the total number of atherogenic and anti-atherogenic lipid particles in plasma, and it may be a better marker of CVD risk than other routine lipid measurements such as LDL cholesterol and HDL cholesterol [9, 30]. There is substantial evidence from epidemiological studies suggesting that a high apoB/A-I ratio is better than any of the cholesterol parameters as a risk marker of future CVD [2, 7, 26, 31–33]. However, the routine clinical use of apolipoproteins has been argued against so far because the apolipoprotein assays are not as widely available relative to standard lipid fractions. Moreover, the therapeutic cutoff points for apolipoproteins are not clearly defined. Therefore, the importance of measuring serum cholesterol lies in that the replacement of cholesterol measurement with apolipoprotein measurements may cause some confusion in clinical practice [8].

CAC, measured using computed tomography, is a noninvasive method for assessing the burden of coronary atherosclerosis. The amount of CAC correlates closely with the amount of atherosclerosis, and this allows improved risk prediction beyond clinical variables in the general population [34]. The extent of CAC is directly proportional to increased cardiovascular event rates [35]. Asymptomatic persons without CAC (scores of zero) are shown to have a very low risk of future cardiovascular events [20]. In a large cohort of asymptomatic men and women, the coronary events were significantly increased in four categories of calcium scores (0, 1–99, 100–399, and ≥400) [20, 36]. The patients with mild RI showed significantly increased coronary calcium score and plaque burden compared to patients with normal kidney function [37]. Our data also revealed that the participants with mid RI showed higher CAC than the participants with normal kidney function (data not shown). To detect subclinical atherosclerosis in a relative health population, the present study defined CAC as an above-zero score.

Impaired renal function is associated with disturbances in lipoprotein metabolism, which results in dyslipidemia and the accumulation of atherogenic particles [10, 11]. Serum apolipoproteins have been shown to significantly associate with eGFR. While apoB showed only inconsistent associations, a higher apoB/A-I ratio was significantly associated with a lower eGFR. Potential confounders such as hypertension, diabetes, and dyslipidemia were addressed, although these were adjusted in the multivariate model [12]. A trend toward significance between worse kidney function and apoB was shown in diabetes patients without CVD, however, this association weakened after further adjustment [17]. The association of the apoB/A-I ratio with coronary heart disease incidence in individuals with CKD, which was defined as an eGFR of 15 to <60 mL/min/1.73 m^2^, was similar to that observed for other lipid measurements in the population-based cohort study. Although CKD is associated with higher ratios of apoB/A-I, the association of CKD with coronary heart disease was similarly attenuated [9].

The strengths of this study include a large healthy population who were underwent a routine heath examination. Thus far, the associations between apolipoproteins and CVD risk have been analyzed in patients with CKD who have CVD risk factors such as hypertension or diabetes or are taking medications indicating a comorbid condition [9]. Hypertension, diabetes, and dyslipidemia are common risk factors for atherosclerosis and may be associated with apolipoprotein levels. We, therefore, attempted to minimize confounding by these factors through extensive exclusion criteria for these confounding factors in the statistical analyses. Moreover, we excluded any evidence of CKD, defined as an eGFR <60 mL/min/1.73 m^2^ or the presence of proteinuria. The present study investigated the effect of apolipoproteins on CAC in the early stage of renal disease, which was defined as mild RI.

We also excluded participants with measured SBP ≥140 mmHg, diastolic blood pressure ≥90 mmHg, or glycosylated hemoglobin ≥6.5% during the visit. In our results, the association between lipid parameters and CAC was found to differ according to kidney function status. In participants with normal kidney function, the apoB/A-I ratio and LDL-cholesterol level were significantly associated with CAC after adjusting other coronary risk factors. Contrary to this, neither the apoB/A-I ratio nor other conventional lipid measurements, such as HDL-cholesterol or LDL-cholesterol levels, showed any significant association with CAC in the participants with mild RI. In the ROC analysis, the AUC of the apoB/A-I ratio was significantly higher for predicting CAC only in the participants with normal kidney function, but the AUC of the apoB/A-I ratio in the participants with mild RI was 0.536.

We can speculate about the potential causes that could explain these discordant findings. Vascular calcification can be caused by inflammation, metabolic disturbances, and genetic factors [38–40]. In patients with CKD, vascular disease has been known to result from atherosclerosis and arteriosclerosis. The higher prevalence of atherosclerosis in patients with CKD than in the general population may be caused by endothelial damage mediated by reactive oxygen species, activation of the renin-angiotensin-aldosterone system, and elevated levels of inflammatory mediators. Arteriosclerosis has been also known in CKD to lead to increased arterial stiffness [40–42]. Abnormal concentrations of atherogenic lipoproteins and lipoprotein subfractions beyond LDL-cholesterol have been also suggested to be associated with the incidence of CAC in CKD, including mild impairments in kidney function [43]. Therefore, the association between mild RI and these possible pathologic processes for CAC might be considered. However, the precise mechanism should be elucidated.

This study has several limitations. First, our study was retrospective cross-sectional in design. Therefore, the precise relationship between apolipoproteins and CAC by kidney function remains unclear. Prospective studies should be conducted to determine whether the association between apolipoproteins and CAC may differ based on kidney function. Second, the findings are limited by the inherent problems with the measurement of serum creatinine, which is dependent on muscle mass, generation, and tubular secretion of creatinine [44, 45]. Third, participants were relatively healthy, and only small percentages had low-to-normal kidney function, although a large number of patients was analyzed. Finally, diagnostic coronary angiography could not be checked to confirm the relationship between the apolipoproteins and the presence of CAC.

In conclusion, the apoB/A-I ratio was significantly correlated with CAC in a healthy population. However, this association appears to differ according to kidney function. Although the measurement of apolipoproteins is a useful parameter for predicting CVD, this should be interpreted carefully, especially in individuals with mild RI.

## Supporting information

S1 Table Characteristics of study participants and post hoc analysis according to kidney function and CAC status. (PDF)
