# Supplementary material for: The association between the apolipoprotein B/A-I ratio and coronary calcification may differ depending on kidney function in a healthy population
Source: PLoS One. 2017 Sep 28;12(9):e0185522. doi: 10.1371/journal.pone.0185522 (PMC5619778; doi:10.1371/journal.pone.0185522)
Supplement: S1 Table — (PDF) [file pone.0185522.s001.pdf]

S1 Table. Characteristics of study participants and post hoc analysis according to kidney function and CAC status

Clinical variables	Normal kidney function & no CAC (1)	Normal kidney function & CAC (2)	Mild RI & no CAC (3)	Mild RI & CAC (4)	Overall *P*-value	Post-hoc *P*-value (Bonferroni corrected *P*-value)					
						(1) vs (2)	(1) vs (3)	(1) vs (4)	(2) vs (3)	(2) vs (4)	(3) vs (4)
Age	49.1 ± 8.6	55.5 ± 7.7	54.7 ± 8.8	62.2 ± 8.9	< 0.001	< 0.001	< 0.001	< 0.001	> 0.999	< 0.001	< 0.001
Sex					< 0.001	< 0.001	0.064	< 0.001	< 0.001	0.320	0.002
Female	416 (38.48)	24 (8.92)	88 (30.34)	24 (15)							
Male	665 (61.52)	245 (91.08)	202 (69.66)	136 (85)							
BMI (kg/m^2)	23.6 ± 3.3	24.3 ± 2.9	24 ± 2.9	24.7 ± 2.9	< 0.001	0.005	0.286	0.001	> 0.999	> 0.999	0.225
SBP (mmHg)	120.2 ± 11.8	123.4 ± 11.4	120.1 ± 12	122.9 ± 11.6	< 0.001	< 0.001	> 0.999	0.043	0.007	> 0.999	0.105
Fasting glucose (mg/dL)	95.2 ± 13.4	101.1 ± 15.9	96.8 ± 12.4	100.7 ± 14.8	< 0.001	< 0.001	0.531	< 0.001	0.001	> 0.999	0.021
eGFR (mL/min/1.73m^2)	103.7 ± 7.9	100.1 ± 6.9	81.6 ± 6.5	80.4 ± 7	< 0.001	< 0.001	< 0.001	< 0.001	< 0.001	< 0.001	0.720
HDL-cholesterol (mg/dL)	50.9 ± 13.3	47.6 ± 10.9	50.2 ± 12.8	48.8 ± 12.5	0.001	0.001	> 0.999	0.312	0.1	> 0.999	> 0.999
LDL-cholesterol (mg/dL)	127.9 ± 33.9	134.7 ± 35.3	137.1 ± 32.6	131.8 ± 33.2	< 0.001	0.018	< 0.001	> 0.999	> 0.999	> 0.999	0.671
Apo A-I (mg/dL)	143.2 ± 24.9	138.5 ± 20.8	142.8 ± 23.6	142.1 ± 25.9	0.038	0.024	> 0.999	> 0.999	0.205	0.809	> 0.999
Apo B (mg/dL)	99.8 ± 24.7	107.5 ± 23.6	100.7 ± 23.2	103.5 ± 23.9	< 0.001	< 0.001	> 0.999	0.436	0.006	0.602	> 0.999
Apo B/ A-I ratio	0.72 ± 0.23	0.8 ± 0.22	0.73 ± 0.22	0.76 ± 0.24	< 0.001	< 0.001	> 0.999	0.331	0.000	0.599	> 0.999
CAC score	0 ± 0	102.97 ± 186.11	0 ± 0	154.77 ± 272.4	< 0.001	< 0.001	> 0.999	< 0.001	< 0.001	< 0.001	< 0.001
Current smoker					0.035	0.383	0.395	> 0.999	0.02	> 0.999	0.876
No	845 (78.17)	196 (72.86)	241 (83.1)	124 (77.5)							
Yes	236 (21.83)	73 (27.14)	49 (16.9)	36 (22.5)							

Data are presented as mean ± standard deviation or number (percentage) of subjects. CAC, coronary artery calcification; RI, renal insufficiency; BMI, body mass index; SBP, systolic blood pressure; eGFR, estimated glomerular filtration rate; HDL, high-density lipoprotein; LDL, low-density lipoprotein; apo, apolipoproteins. Normal Mild RI was defined as an eGFR of 60-90 mL/min/1.73 m^2. A comparative analysis of four groups was performed with analysis of variance test. *P*-values of post-hoc analysis were corrected by Bonferroni correction.
